# A Case Review Series of Christiana Care Health System’s Experience with Negative Pressure Wound Therapy Instillation

**DOI:** 10.7759/cureus.865

**Published:** 2016-11-07

**Authors:** Robert Felte, Kathy E Gallagher, Glen H Tinkoff, Mark Cipolle

**Affiliations:** 1 Department of Surgery, Surgical Critical Care Wound Service, Christiana Care Health System; 2 Director, Surgical Service Line Outcomes, Department of Surgery, Christiana Care Health System

**Keywords:** negative pressure wound, wound care, npwt with instillation, negative pressure wound therapy with instillation

## Abstract

Acute and chronic wounds afflict a multitude of patients to varying degrees. Wound care treatment modalities span the spectrum of technological advancement and with that differ greatly in cost. Negative pressure wound therapy (NPWT) can now be combined with instillation and dwell time (NPWTi-d). This case review series of 11 patients in a community hospital setting provides support for the utilization of NPWTi-d. Additionally, current literature on the use of NPWTi-d in comparison to NPWT will be reviewed.

We highlight three specific cases. The first case is a 16-year-old male who was shot in the left leg. He suffered a pseudoaneurysm and resultant compartment syndrome. This required a fasciotomy and delayed primary closure. To facilitate this, NPWTi-d was employed and resulted in a total of four operative procedures before closure 13 days after admission. Next, a 61-year-old uncontrolled diabetic female presented with necrotizing fasciitis of the lower abdomen and pelvis. She underwent extensive debridement and placement of NPWTi-d with Dakin’s solution. A total of four operative procedures were performed including delayed primary closure six days after admission. Finally, a 48-year-old female suffered a crush injury with internal degloving. NPWTi-d with saline was utilized until discharge home on postoperative day 12.

NPWTi-d, when compared to NPWT, has been reported to lead to a decrease in time to operative closure, hospital length of stay, as well as operative procedures required. The cost-benefit analysis in one retrospective review noted a $1,400 savings when these factors were taken into account.

This mode of wound care therapy has significant benefits that warrant the development of a prospective randomized controlled trial to further define the improvement in quality-of-life provided to the patient and the reduction of potential overall healthcare costs.

## Introduction

Negative pressure wound therapy (NPWT) has been showing great promise in its ability to facilitate wound healing which indirectly decreases healthcare resource utilization while improving patients’ quality of life [1]. Based on a retrospective review, the addition of negative pressure wound therapy with instillation and dwell time (NPWTi-d) to our standard of care may result in a positive cost-benefit ratio. As such, our responsibility to the global health system elicits our need to report our results for critique and peer review so that we may continue to advance patient care. We present our utilization of NPWTi-d and highlight three, particularly interesting cases.

## Case presentation

### Case 1

A 16-year-old male presented as a trauma-alert status, post-gunshot wound through-and-through to the right groin and into the left proximal thigh. He was stabilized and taken for CT imaging. Active extravasation of contrast was seen from the left profunda. As a result, the patient was taken to the interventional radiology suite where he underwent successful coil embolization of a pseudoaneurysm involving the medial branch of the left profunda.

**Figure 1:**
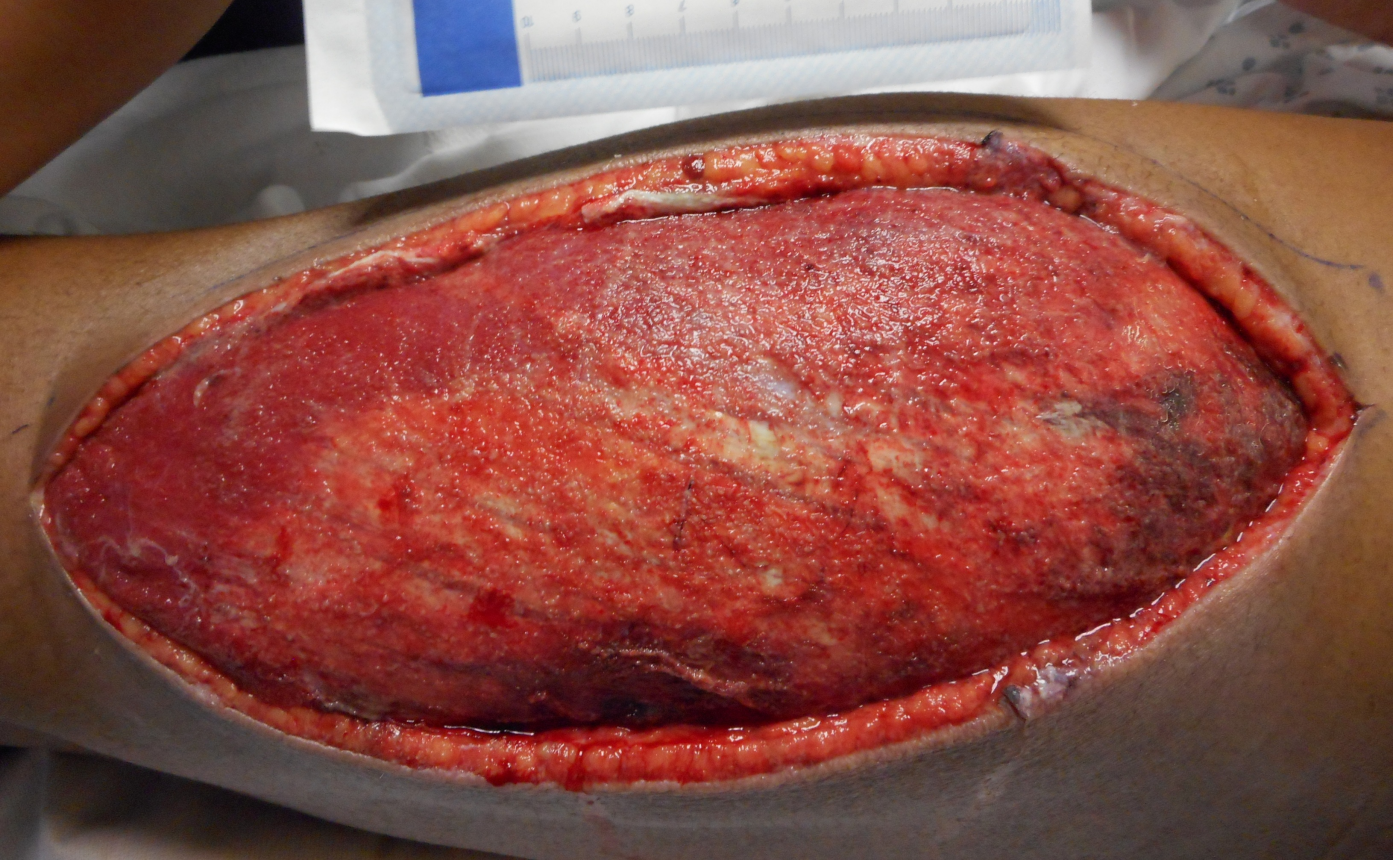
Compartment Syndrome 10 cm x 27 cm lateral lower extremity fasciotomy after the development of compartment syndrome.

Over the next few hours, he developed features concerning for compartment syndrome. He was taken to the operating room, and a left lateral thigh fasciotomy was performed. Wound margins measured 10 x 27 cm. In order to facilitate compartment checks, this was initially managed with wet-to-dry dressings, and can be noted in the image below in Figure 1.

On postoperative day 3, the patient was taken back to the operating room for a wound washout and placement of an NPWTi-d device. Pressure was maintained at 125 mmHg, and saline solution was infused every four hours. Another OR trip was planned for postoperative day 7 for wound washout and to change the negative pressure wound therapy device.

By postoperative day 13, the patient’s wound measured 5 x 15 cm. He was taken to the operating room a fourth and final time for delayed primary closure. Throughout his hospital course, the patient continued to work well with physical and occupational therapy. He was finally discharged home on postoperative day 14 with excellent regain of function of the left leg.

### Case 2

Next, a 61-year-old female with a history of uncontrolled diabetes presented to our emergency department with an altered mental status and generalized abdominal pain. A CT scan of the abdomen and pelvis showed extensive subcutaneous emphysema of the anterior abdominal wall extending into the perineum. She was taken emergently to the operating room for excisional debridement and washout. The wound extended from left of the midline at the level of the umbilicus all the way down to the pubic symphysis with a separate counter incision at the labia major. This was initially managed with wet to dry dressing changes.

**Figure 2:**
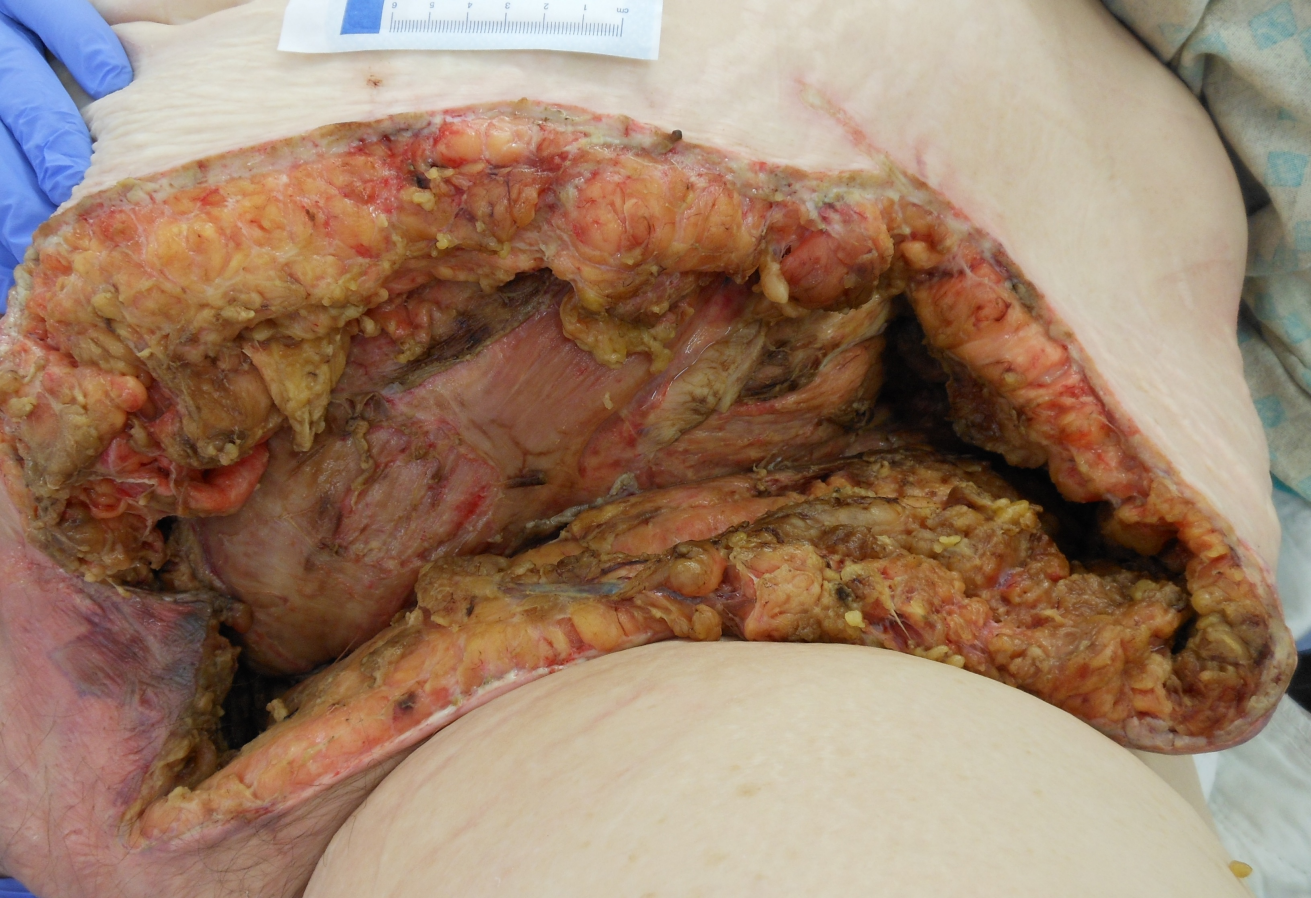
Necrotizing Fasciitis Before the Second Operative Debridement

**Figure 3:**
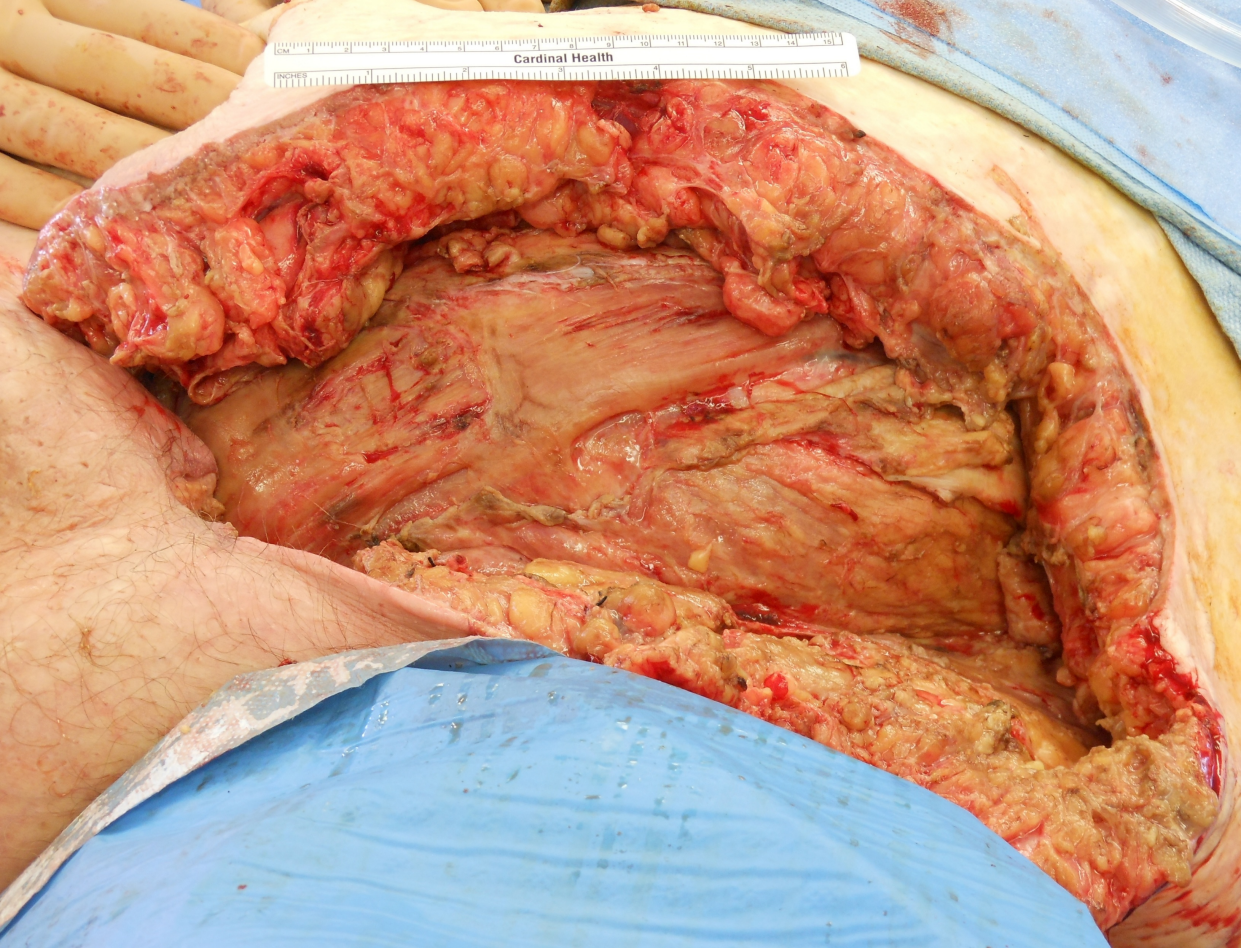
Necrotizing Fasciitis Post-Debridement

On postoperative day 3, the patient was taken back to the operating room for debridement, washout, and placement of an NPWTi-d device. Suction was maintained at a pressure of 125 mmHg, and Dakin’s solution was infused every four hours. Figure 2 and Figure 3 shown below represent the wound prior to NPWTi-d initiation before and after the second operative debridement, respectively.

Postoperative day 6, she returned to the operating room for a third and final time for delayed primary closure of the entire complex wound. Prevena^TM^ Incisional Management System by Acelity and two Blake drains were used to contain the drainage at the closure site.

The patient made satisfactory progress throughout the hospital course. By postoperative day 13, the wound-management system was ready to be taken down. She was discharged to an extended care facility in stable condition.

### Case 3

The final case is a 48-year-old female who was run over by a car and sustained a soft-tissue crush injury to her left leg with internal degloving. Although her skin opening was 4 x 6 cm, the wound extended circumferentially around the wound with undermining at least 4 cm and tracking halfway down the calf. The eschar was debrided with electrocautery expressing serosanguinous fluid. NPWTi-d foam was inserted deep into the cavity,and 50 ml normal saline every six hours for ten-minute soaks were initiated at 125 mmHg pressure.       

**Figure 4:**
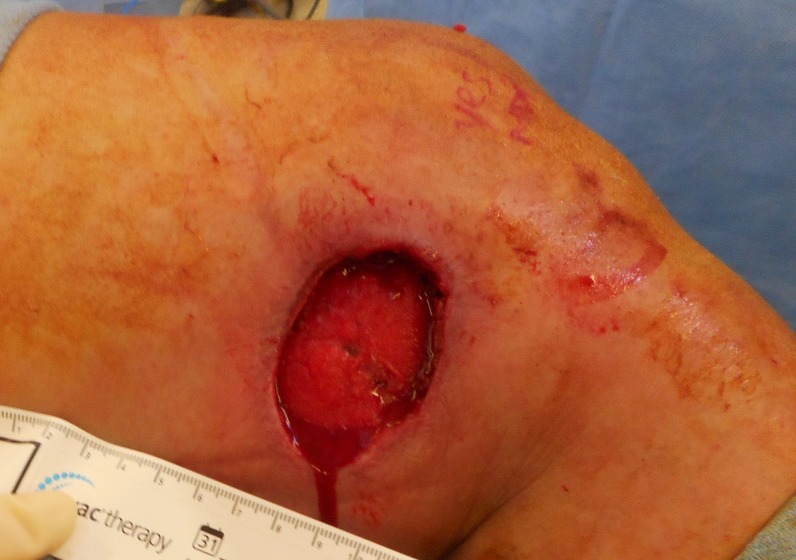
Postoperative Day 2

On postoperative day 2, she returned to the operating room for a washout of the 4 x 5.5 cm opening and 10 x 20 cm extending underneath into the subcutaneous pre-fascial space as noted in Figure 4 below. Granulation was visualized without signs of infection. NPWTi-d was continued.

**Figure 5:**
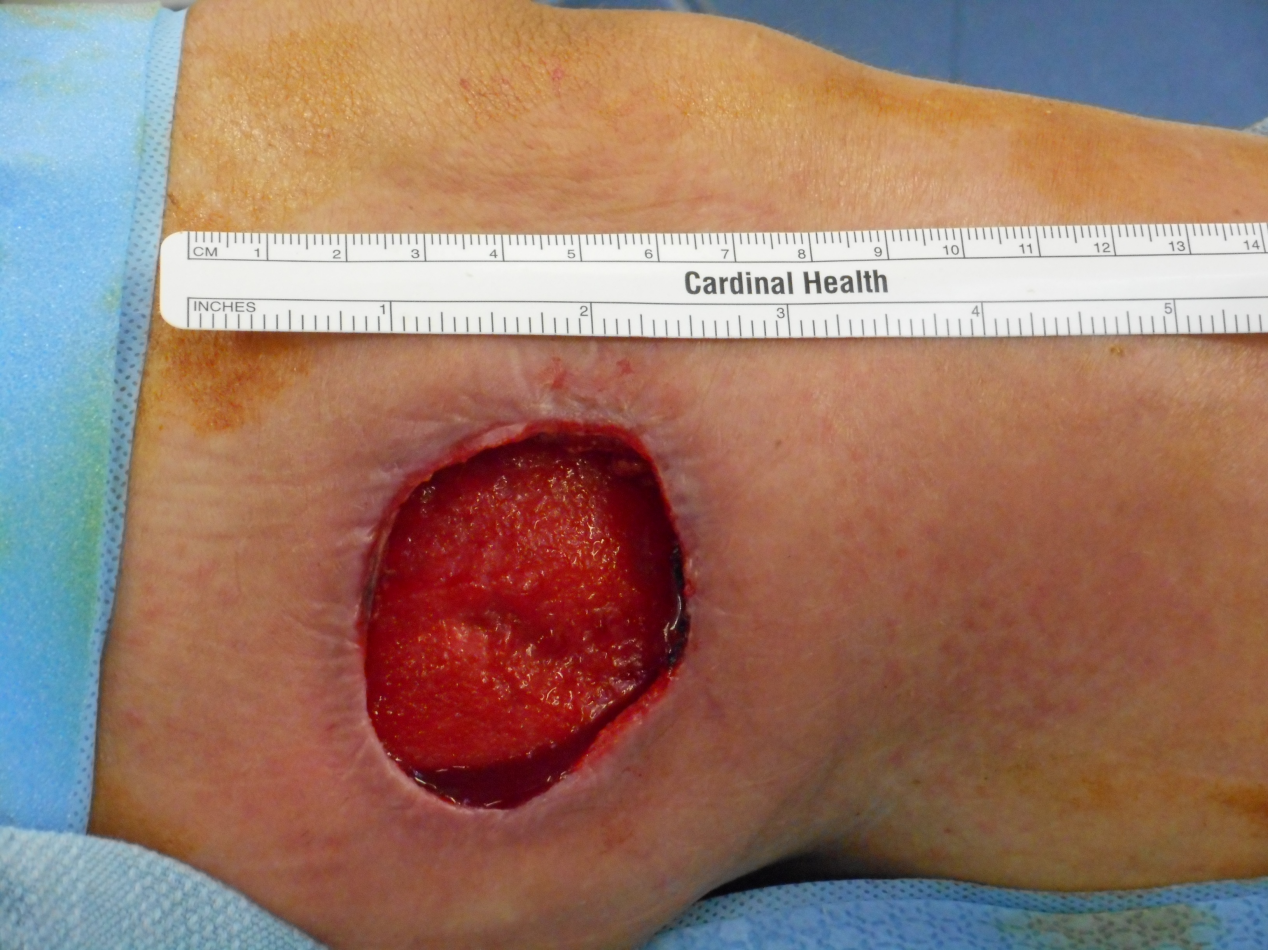
Postoperative Day 5

On postoperative day 5 in the operating room, the wound was again washed out. The extension into the underlying tissues was found to be significantly reduced at 7.5 x 13.5 cm as noted below in Figure 5. NPWTi-d with saline instillation was continued.

**Figure 6:**
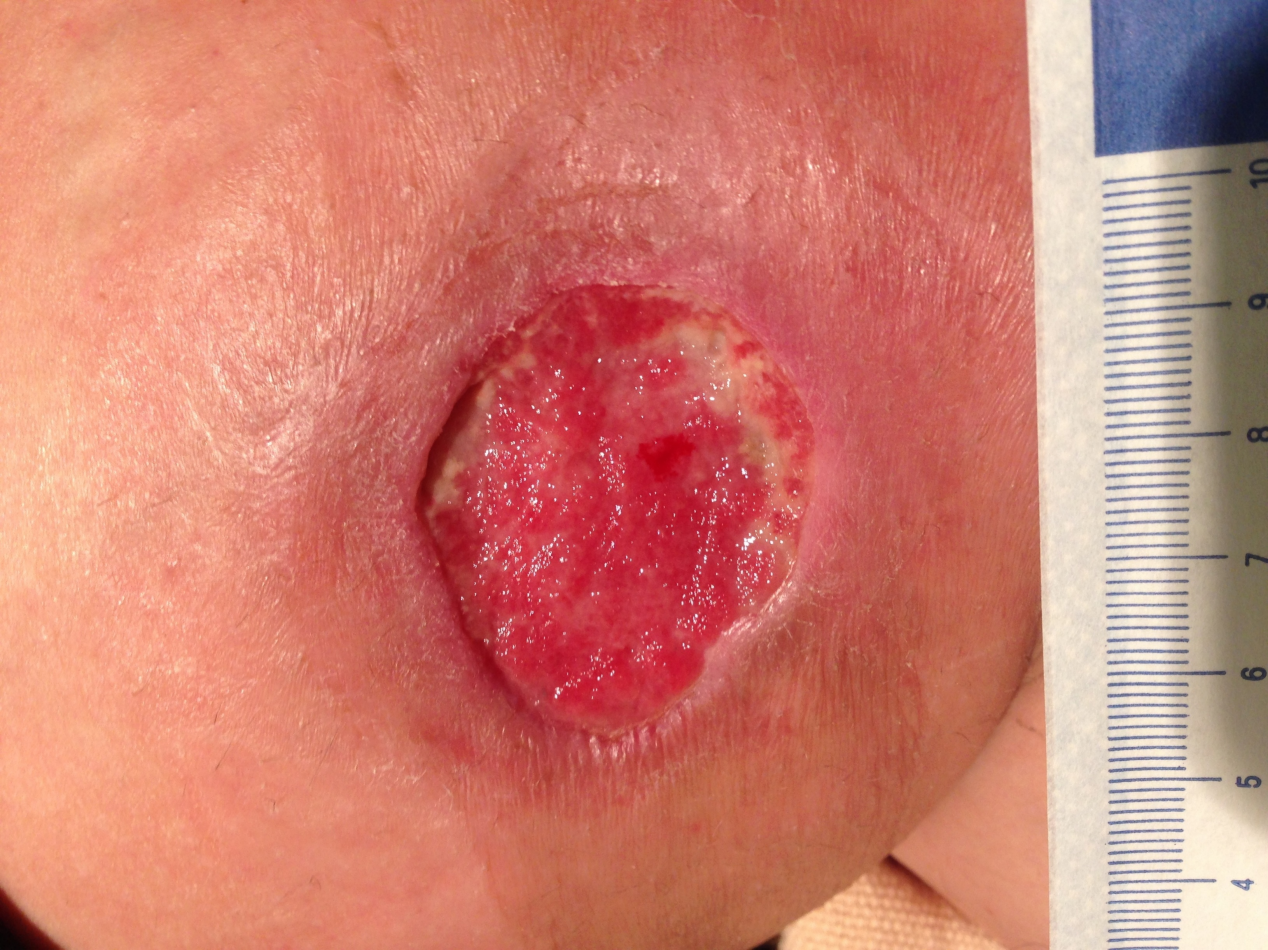
5-Week Postoperative Follow-Up

On postoperative day 9 in the operating room, the wound was washed out finding healthy granulation tissue without infection. The opening was 3.5 x 5 cm, yet now 5 x 6.5 cm extension into the subcutaneous space was noted. Although she could have then been managed as an outpatient as evidenced in Figure 6, her socioeconomic status delayed her initial discharge. NPWTi-d was taken advantage of until postoperative day 12 when she was converted to traditional NPWT for discharge home.

The patients agreed to participate and were explained the nature and objectives of this study, and informed consents were formally obtained. No references to the patients' identities were made at any stage during data analysis or in the report.

## Discussion

At our institution, we have a total of 11 cases in whom NPWTi-d has been utilized. Two of these cases are currently being managed and approaching closure. We have noted increased granulation tissue at a more precipitous rate than with NPWT alone. This has resulted in a reduced wound volume with undermining of the wound beds filling in more rapidly. Brinkert et al., in 2013 published similar findings [2]. Their prospective case series evaluated outcomes in 131 patients with complex wounds treated by NPWTi-d. An instillation dwell time of 10 minutes and NPWT between 4-12 hours was employed in their study [2]. Our instillation dwell time and NPWT has been standardized across our cases. Patients undergo NPWTi-d for 10 minutes followed by 4 hours of NPWT at -125 mmHg continuously with Dakin’s or normal saline. The amount of instillate in milliliters equates to approximately 20% of the wound area in square centimeters.

As an acute care service, we take on many trauma patients as well as individuals with multiple comorbidities. Admitting diagnoses and mechanisms range from necrotizing fasciitis, Fournier’s gangrene, and postoperative abdominal wall abscesses to lacerations and compartment syndrome status post-crush injuries and gunshot wounds. These types of injuries and disease processes lend themselves to a variety of colonization possibilities. Some we have encountered include Enterococcus faecalis, Enterobacter sakazakii, Eikenella corrodens, Haemophilus species, methicillin-resistant Staphylococcus aureus, Escherichia coli, Candida albicans, Corynebacterium species, and Actinomyces species. In cases where the patient presents with a complex wound, either contaminated on tissue culture or grossly infected on clinical physical examination, we have used Dakin’s Quarter Strength (sodium hypochlorite) 0.125% for instillation therapy after thorough surgical debridement. With the Dakin’s therapy for the complex wounds as well as  normal saline for clean wounds, our patients were able to be closed 36.5 days on average in delayed primary fashion, skin graft, or with muscle flap advancement.  

**Figure 7:**
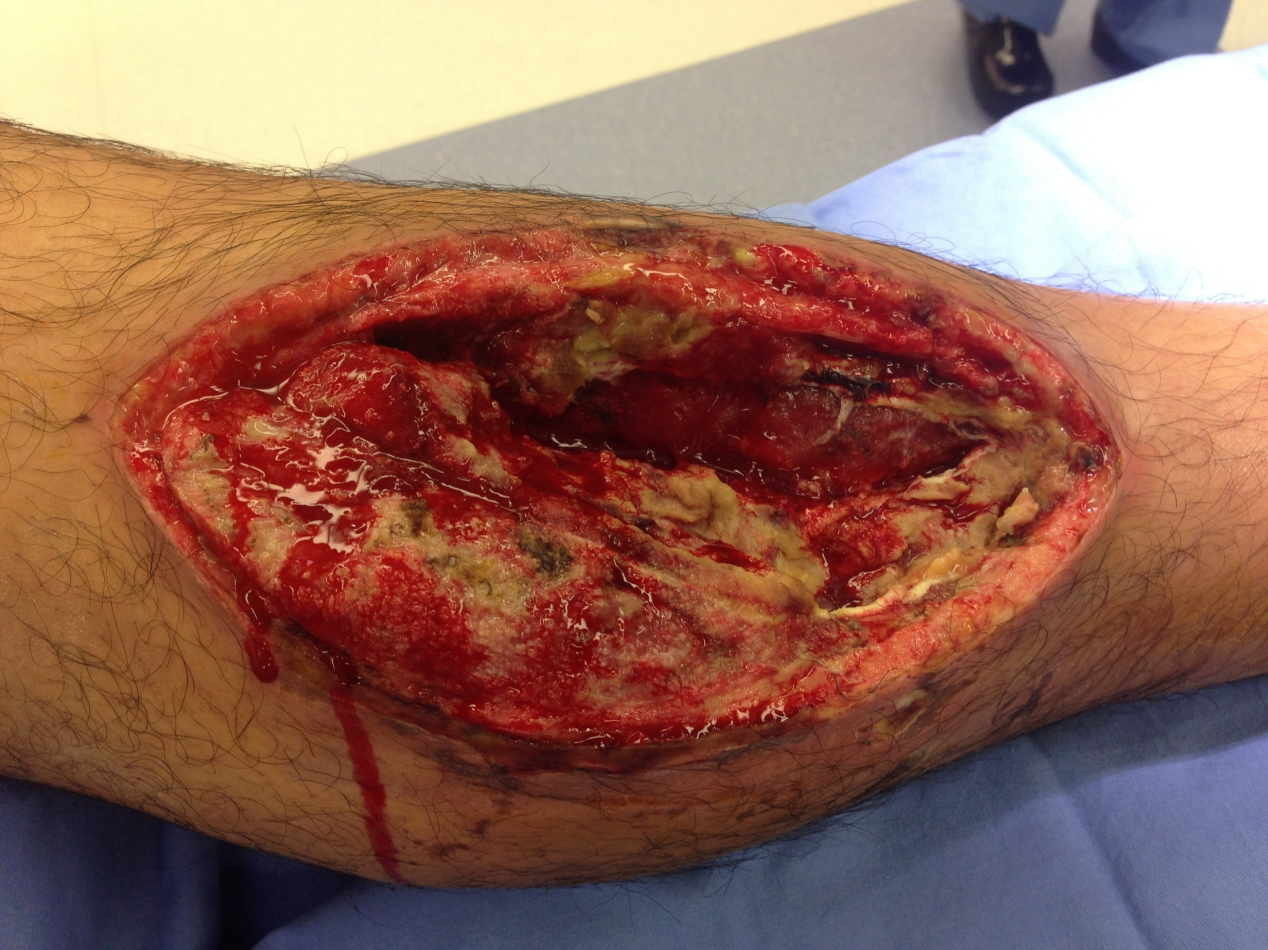
Before Dakin's NPWTi-d Enterococcus faecalis, Enterobacter sakazakii, Eikenella corrodens, and Haemophilus species colonizing an infected fasciotomy site, status post-gun shot to the lower extremity.

Goss et al., tested quarter strength Dakin’s and its efficacy on chronic leg wounds. With their instillation time of 10 minutes followed by 1 hour of NPWT, they were able to show a reduction in colony-forming units (CFU) per gram of tissue culture when compared to NPWT alone. Their results were not statistically significant, and both groups underwent this therapy after sharp debridement; however, they were able to show an overall increase in CFUs in the NPWT group and a decrease in CFUs in the NPWTi-d group. This therapy was continued for a total of 7 days [3]. As noted above, we have begun to use quarter-strength Dakin’s solutions in all patients with necrotizing soft tissue infections or grossly contaminated wounds. Figure 7 depicts a medial lower extremity wound colonized with Enterococcus faecalis, Enterobacter sakazakii, Eikenella corrodens, and Haemophilus species.

**Figure 8:**
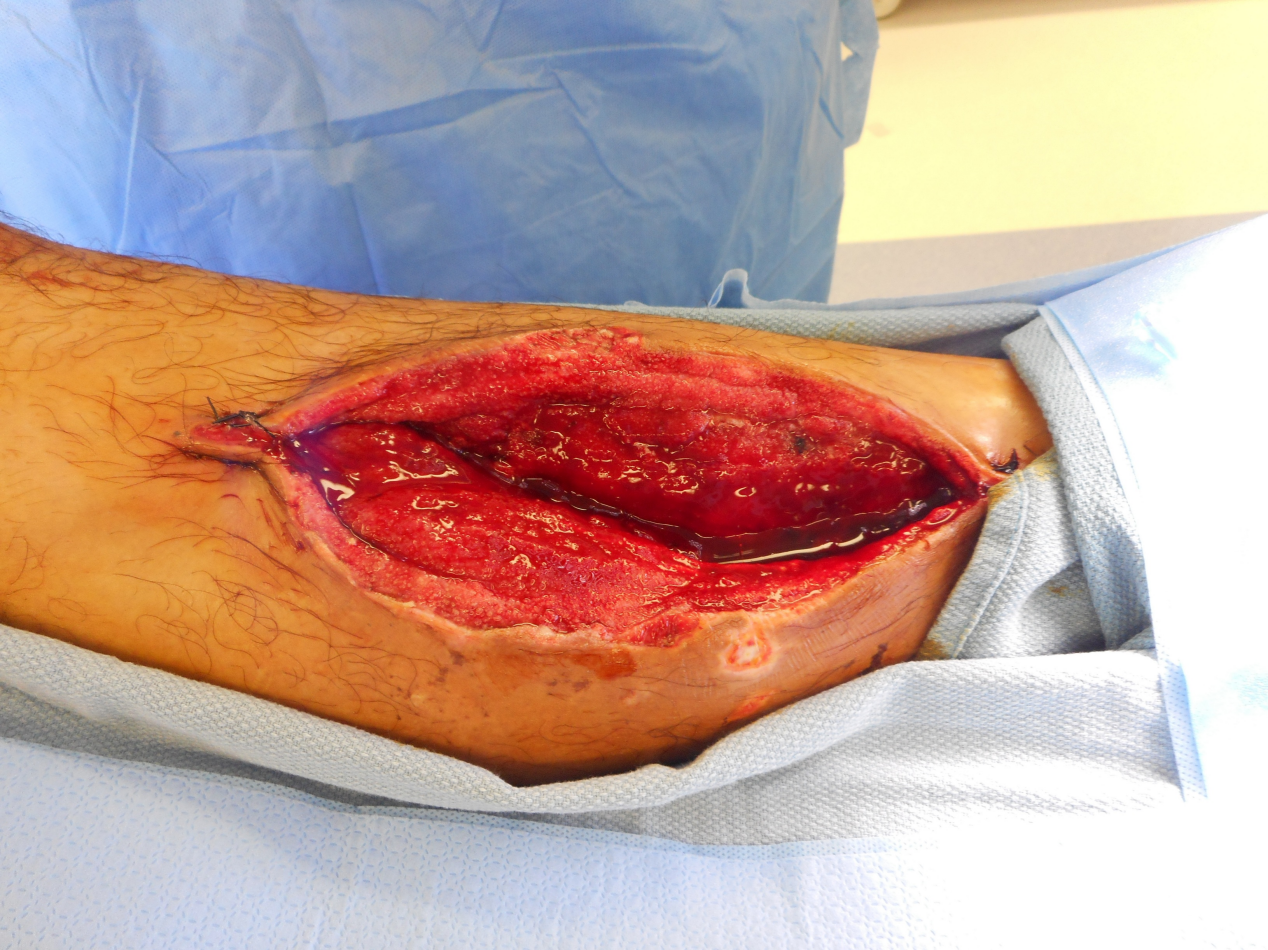
After Dakin's NPWTi-d Fasciotomy site 6 days after Dakin's NPWTi-d.

After 6 days of Dakin’s NPWTi-d, Figure 8 is notable for a clean base with beefy red granulation tissue and measures 22 x 11 x 4 cm.

**Figure 9:**
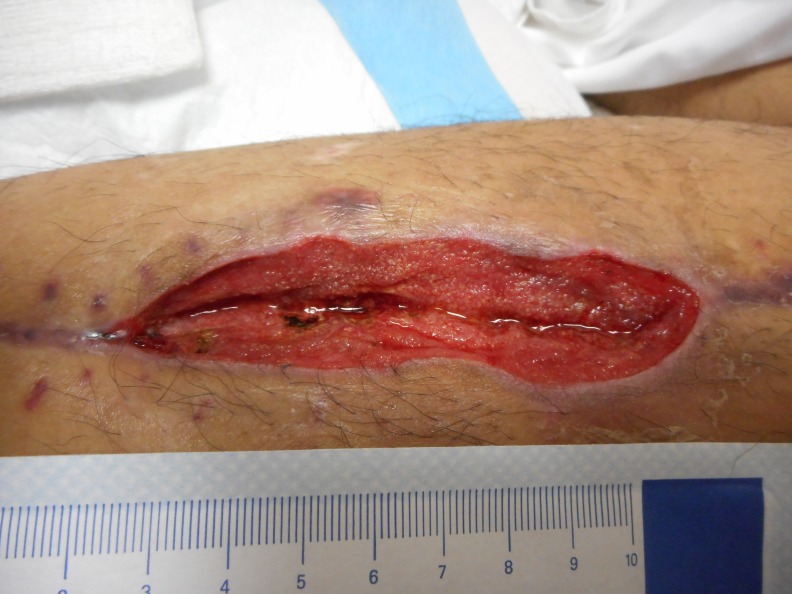
Week 8 Prior to delayed primary closure at week 8 from initial fasciotomy.

NPWT continued for an additional 7 weeks with results shown in Figure 9, prior to delayed primary closure measuring 8 x 2 cm.

Kim et al., compared the outcomes of patients receiving negative pressure wound therapy with instillation of Prontosan--a wound irrigation solution containing purified water, undecylenamidopropyl Betaine 0.1%, and polyamino propyl biguanide 0.1%--with two groups of instillation times, 6 and 20 minutes, to historical controls who only received negative pressure wound therapy. Variables analyzed included the number of operative procedures, length of hospital stay, time to final surgical procedure, percentage of wounds that were surgically closed before discharge, percentage of wounds that remained closed 30 days after discharge, and reduction in microorganisms. Their results showed a statistical difference in favor of instillation therapy in both the 6- and 20-minute groups when compared to the NPWT for the number of operative procedures and the time to final surgical procedure. The decrease in length of hospital stay was only statistically significant when compared to the 20-minute dwell time [4]. These findings suggest that instillation therapy not only benefits the patient but also the entire health care system, with potential decreased hospital stays and decreased operating room expenditures. In our patients, we have an average time to closure of 37 days with trips to the operating room averaging 7 per patient as noted in Table 1.

**Table 1:** Patients Undergoing NPWTi-d GSW: gun shot wound, HCV: hepatitis C virus, PSD: poly substance dependance, OA: osteoarthritis, HLD: hyperlipidemia, CVA: cerebral vascular accident, EtOH: alcohol abuse, Tobacco: tobacco abuse, DM: diabetes unspecified, HTN: hypertension, CAD: coronary artery disease, Active: wounds active in delayed primary repair, SSI: surgical site infection lead to secondary intention closure, Saline: normal saline instillation therapy, Dakin's: quarter strength Dakin's solution Patient 1: Case 1 in article, Patient 9: Case 2 in article, Patient 6: Case 3 in article

Patient	Sex	Age	Diagnosis	Location	Trauma	PMH	OR trips	Days to closure	NPWTi	Bacteria
1	M	16	Compartment syndrome	Right distal leg	GSW	Tobacco	6	Active	Saline	N/A
2	M	20	Laceration	Right medial calf	Laceration	Asthma	5	Active	Saline	N/A
3	M	16	Compartment syndrome	Left lateral thigh	GSW	Tobacco	4	13	Saline	Pseudomonas aeruginosa
4	M	24	Delayed primary closure	Abdominal wound	GSW	None	3	7	Saline	Pseudomonas aeruginosa, Escherichia coli
5	M	25	GSW, Compartment syndrome	Right distal leg	GSW	Tobacco	9	63	Dakin's	Enterococcus faecalis, Enterobacter sakazakii, Eikenella corrodens, Haemophilus
6	F	48	Crush injury	Left medial thigh	Pedestrian struck	HCV, Tobacco	4	Secondary intention	Saline	Corynebacterium species, Staphylococcus aureus
7	M	27	Compartment syndrome	Right forearm, Right thigh	Drug OD	Tobacco, PSD	11	SSI	Saline	Klebsiella, Enterococcus, Coagulase neg staph
8	M	51	Abd wall abscess	Infected retrorectus hematoma	VHR	Diverticulitis, OA, HLD, CVA, CVA, EtOH, Tobacco	5	Secondary intention	Saline	moderate anaerobic GPR, rare GNR
9	F	61	Necrotizing fasciitis	Abdomen, Pelvis	No	DM, HTN, Tobacco, EtOH	4	6	Dakin's	No growth
10	F	77	Necrotizing fasciitis	Bilateral flank, Perineum/labia, Bilateral medial thigh	No	Asthma, COPD, HTN, Hypothyroidism	10	27	Saline	Enterococcus faecium, Enterococcus faecalis
11	M	59	Necrotizing fasciitis	Perineum	No	Afib, Cirrhosis, EtOH, Fournier's gangrene, HLD, HTN, CAD	12	103	Dakin's	Escherichia coli, Candida albicans, Staphylococcus spp., Corynebacterium species, Actinomyces species
Average		39					7	37		

We have not compared this to our historical NPWT data; however, Gabriel et al., noted an approximate $1,400 savings per patient in their retrospective analysis of NPWTi-d using saline and polyhexanide compared to NPWT alone [1]. More importantly, these findings that suggest NPWTi-d increases throughput in the hospital system which can translate into a quicker return for the patient back to their previous quality of life.

## Conclusions

The goal of any innovation is to better provide our patients with quality care. How to achieve that quality without added expense for the patient and the overall healthcare system is the hurdle we must overcome. By providing the appropriate environment, NPWTi-d has been shown in the literature to support an increased rate of wound healing. When one considers how many patients are affected by both acute and chronic wounds, the possible impact of this therapy should not be overlooked. NPWTi-d has the opportunity to have a great impact on patient care and healthcare in general. As such, a prospective randomized controlled trial comparing multiple modalities of wound care including NPWTi-d with and without antibacterial adjuncts should be pursued.
